# Supplementary material for: The Role of Hemodynamics through the Circle of Willis in the Development of Intracranial Aneurysm: A Systematic Review of Numerical Models
Source: J Pers Med. 2022 Jun 20;12(6):1008. doi: 10.3390/jpm12061008 (PMC9225067; doi:10.3390/jpm12061008)
Supplement: Supplementary file 1 [file jpm-12-01008-s001.zip › Supplementary S1.pdf]

Supplementary S1: Search strategy

Medline

("Circle of Willis"[Mesh] OR willis*[tiab] OR basilar circulation*[tiab] OR ((cerebral[tiab] OR cerebri[tiab]) AND (circle[tiab] OR circulus[tiab])) OR arterial circle [tiab] OR brain circulation[tiab] OR intracranial circulation[tiab] OR cerebral circulation[tiab] OR basilar circulation[tiab])

AND

("Intracranial Aneurysm"[Mesh] OR aneurysm* [tiab])

AND

("Hemodynamics"[Mesh] OR hemodynamic* [tiab] OR haemodynamic*[tiab] OR Shear stress[tiab] OR Laminar flow[tiab] OR Turbulent flow[tiab] OR Computational fluid dynamic*[tiab] OR CFD[tiab] OR Intra-aneurysmal flow[tiab] OR intraaneurysmal flow[tiab] OR flow velocity*[tiab] OR flow rate*[tiab])

NOT

("Animals"[Mesh] NOT "Humans"[Mesh])

Embase

('brain circulus arteriosus'/exp OR (willis* OR ((circle OR circulus OR circulation) NEAR/3 cerebr*) OR ‘basilar circulation’ OR ‘arterial circle’ OR ‘brain circulation’ OR ‘intracranial circulation’):ab,ti)

AND

('intracranial aneurysm'/exp OR 'aneurysm*':ab,ti)

AND

('hemodynamics'/exp OR (hemodynamic* OR haemodynamic* OR 'shear stress' OR 'laminar flow' OR 'turbulent flow' OR 'computational fluid dynamic*' OR cfd OR 'intra-aneurysmal flow' OR ‘intraaneurysmal flow’ OR ' flow velocity' OR 'flow rate'):ab,ti)

NOT

('animal'/exp NOT 'human'/exp)

Web of Science

TS= (willis* OR (cerebr* NEAR/3 (circle OR circulus OR circulation)) OR “basilar circulation” OR “arterial circle” OR “brain circulation” OR “intracranial circulation”)

AND

TS= aneurysm*

AND

TS= (hemodynamic* OR haemodynamic* OR “shear stress” OR “laminar flow” OR “turbulent flow” OR “computational fluid dynamic*” OR cfd OR “intra-aneurysmal flow” OR “intraaneurysmal flow” OR “flow velocity” OR “flow rate”)
