# Supplementary material for: The Role of Hemodynamics through the Circle of Willis in the Development of Intracranial Aneurysm: A Systematic Review of Numerical Models
Source: J Pers Med. 2022 Jun 20;12(6):1008. doi: 10.3390/jpm12061008 (PMC9225067; doi:10.3390/jpm12061008)
Supplement: Supplementary file 1 [file jpm-12-01008-s001.zip › Supplementary S2.pdf]

Supplementary S2: Studies excluded due to language

1. Gonzalez-Llanos F, Pascual JM, Roda JM. Anatomic and hemodinamic, study of the anterior communicating artery complex. Neurocirugia. 2002;13(4):285-298.

2. Grigor'eva E V, Krylov V V. Assessment of cerebral hemodynamics using computed tomography perfusion in patients with intracranial aneurysms. Vestn Rentgenol Radiol. 2015;(2):5-14.

3. Todaro CA, Arena O, Fontana RA, et al. Transcranial doppler monitoring in cavernous sinus lesion. Pre and post operative evaluation of two cases. Riv di Neurobiol. 1997;43(2-3):137-140.

4. Qiu X, Fei Z, Wang W, Cao Z. Numerical analysis on hemodynamics of cerebral aneurysm clip. J Biomed Eng. 2012;29(1):102-106,111.
